# Empowering consumers to purchase safe ready-to-eat chicken from street restaurants in Ouagadougou, Burkina Faso: impact of a multi-media behavior change campaign

**DOI:** 10.1038/s41598-024-76123-4

**Published:** 2024-10-21

**Authors:** Donya S. Madjdian, Marcel van Asseldonk, Elise F. Talsma, Michel Dione, Guy Ilboudo, Kristina Roesel, Delia Grace, Theodore J.D. Knight-Jones, Emely de Vet

**Affiliations:** 1 Consumption and Healthy Lifestyles, Wageningen University & Research, P.O. Box 8130, Wageningen, 6700EW the Netherlands; 2 Wageningen Economic Research , Wageningen, the Netherlands; 3 Division of Human Nutrition & Health, Wageningen University & Research, Wageningen, the Netherlands; 4 International Livestock Research Institute (ILRI), Ouagadougou, Burkina Faso; 5 International Livestock Research Institute (ILRI), Nairobi, Kenya; 6 Natural Resources Institute, University of Greenwich, Chatham, United Kingdom; 7 University College Tilburg, Tilburg University, Tilburg, The Netherlands

**Keywords:** Burkina Faso, Food safety, Consumer behavior, Behavior change, Informal markets, Consumer campaign, Public health, Disease prevention

## Abstract

Foodborne disease resulting from food sold at urban informal markets is a major public health challenge in Sub-Saharan Africa. This study assessed the impact of an innovative nine-months multi-media campaign engaging a key influencer, aimed at empowering consumers to choose safer ready-to-eat chicken meat at informal street restaurants in Ouagadougou, Burkina Faso. A two-wave panel study assessed associations between recall of TV, radio, billboard, and social media advertisements, and self-reported behavior regarding purchasing and consumption of ready-to-eat chicken at outlets, consumer intentions, knowledge, attitudes, norms, and agency. A panel of randomly selected adult consumers (*n* = 852) were interviewed pre- and post-campaign, 12 months apart. 60% recalled at least one of the campaign channels when prompted. Mixed-effects models showed associations between prompted recall and feeling better informed about food safety (aOR 1.449) and increased knowledge (0.132 unit increase on total score). Social media recall was associated with higher perceived access to information (aOR 1.449) and knowledge. Billboard recall increased odds of higher perceived health benefits of paying attention to food safety behaviors when purchasing chicken (aOR 2.046). TV ad recall was associated with a 0.159 unit decrease in the gap between consumers’ intentions and behavior. An engaging consumer food safety multimedia campaign that engages key influencers improved food safety awareness and knowledge, ultimately supporting consumers to choose safer chicken at markets.

**Supplementary Information:**

The online version contains supplementary material available at 10.1038/s41598-024-76123-4.

## Introduction

A critical component of food and nutrition security, food safety is a global, but often neglected challenge with far-reaching implications for public health, nutrition, economic, and societal development. In low-and middle-income countries, foodborne diseases cost USD115 billion annually^1^. Sub-Saharan Africa has the highest per capita burden. The costs associated with lives lost and human sickness due to foodborne disease are estimated between USD20 and 40 billion^2,4^. In Africa, food safety concerns are especially relevant due to its rapidly growing population, urbanization, and diversification of food production systems that are chaning dietary patterns^5^. This includes a shift towards eating outside the home and consuming street foods more frequently^6^. The domestic, informal market sector plays a key role in the local economy by providing affordable and accessible food to a significant portion of the urban population ^7^. However, informal markets are challenged by limited infrastructure and regulatory oversight, lack of access to safe water, sanitation, waste disposal systems, and environmental pollution. Consequently, a large share of food safety risks and foodborne disease is attributed to food sold at these markets^8,10^.

In Burkina Faso, the estimated foodborne disease burden in terms of Disability Adjusted Life Years (DALY) is 328,064 with many food-hazards attributed to animal-source products including chicken meat^2^. The poultry sector is key to the economic development of Burkina Faso: chicken meat is an important protein source in local diets and regularly consumed at home and out of the home, with an estimated per capita consumption of approximately eight kilos of poultry meat per year^11^, with much higher consumption in Ouagadougou, the capital. Among the urban population, mostly adult consumers from medium and higher income households, flamed or grilled chicken are particular popular street foods purchased from the many open-air street restaurants called *maquis*^12^. Despite the popularity of ready-to-eat chicken, there are notable food safety risks associated with its consumption^13,14^. Studies have shown high prevalence of *Salmonella* spp. (up to 90%) and *Campylobacter* spp. (up to 86%) in poultry sold in Ouagadougou^15^. In 2017, poultry meat contaminated with *Campylobacter* spp. and *Salmonella* spp. resulted in 42,600 DALYs, with around one in 50 people falling ill from eating poultry meat due these hazards^16^.

Poor cold chains and unhygienic vendor practices create chicken safety risks, with cross-contamination spreading pathogens from raw chicken to other foods. Live chicken, mostly sourced from outside the city, are often transported by motorcycles to urban markets or roadside traders, jeopardizing the health of the birds^11^. From here chickens are sold to vendors, retailers, or street eateries. Slaughter points in markets or restaurants pose additional risks due to poor hygiene practices by vendors, such as improper handwashing, unclean water, ambient temperatures, unsanitary slaughtering, and dirty surroundings and tools^12^. Firewood-flamed chicken poses higher risks due to shorter grilling times and lower temperatures compared to boiling or deep-frying chicken meat. Grilled chicken served with raw vegetables (e.g., onions, tomatoes) also carries risks due to cross-contamination from raw chicken to uncooked vegetables^17^.

Ensuring food safety in Africa’s urban informal markets is crucial for food security, public health, and preventing foodborne disease. However, in this context, the collective responsibility of actors from farm to fork is often overlooked. While the formal sector’s oversight is somewhat overseen by authorities, little regulatory attention is paid to the informal sector in Africa. Also overlooked is the role that consumers play as agents of change by choosing to preferentially purchase safer food^18^. Although in high-resource contexts, consumer demand drives retailers to ensure food safety to avoid scares impacting sales and reputation^19^, this is not necessarily recognized in African informal food markets.

Mass media communication campaigns have potential to promote consumers’ food safety behavior change through raising awareness and encouraging safer food practices. Such campaigns can influence behavior both directly by targeting individual decision-making and indirectly via social norms. In the context of health promotion, campaigns have helped to remove obstacles to change, promote food safety norms, and create a supportive environment for behavior change^20^. In the domain of food safety, effective strategies have involved emphasizing risk perception and using emotion-based approaches^21^. However, uncertainty pertains to which media delivery channels are best suited in this context^21^. A recent study in Ethiopia assessing the effects of a mass media behavior change campaign to promote the purchase of safe tomatoes in informal markets, has shown promising associations with safer self-reported consumer buying behavior, as well as increases in food safety intentions, knowledge, benefits, norms, and self-efficacy. Household visits and traditional media channels such as TV and radio and billboards appeared most strongly associated with these changes^22^. In Nigeria, however, mass media did not change behaviors or increase awareness of Lassa fever, although it did enhance understanding of hygiene behaviors related to food safety^23^. Behavioral change theories posit that for such campaigns to be effective and drive behavior change, it is essential that key behavioral determinants, such as attitudes, norms, agency, knowledge, skills, and intentions are addressed^24,25^. Generally, while knowledge is important for making informed decisions and understanding risks, adhering to food safety behavior often requires more than knowledge^26^. Consumers must be willing to change unsafe habits, and so their intentions to do so are also key predictors of behavior change. In addition, prevailing norms in society, attitudes towards certain food safety practices, and agency (i.e., perceived decision-making power) determine one’s intention to engage in safe practices. To date, there is a paucity of studies investigating the effects of consumer-focused educational interventions on behavioral drivers^21^, although these preceding individual motives could offer insights for more targeted interventions to promote food safety.

In summary, despite evidence from high-income countries on the potential of consumer-focused interventions to improve food safety behavior, and behavioral drivers such as awareness, knowledge, and intentions^27,30^, little is known on the effects of such interventions in the context of Sub Saharan Africa^21,31^. Moreover, systematic reviews indicate that studies mostly focused on in-home food safety, neglecting behaviors outside the home and in market settings where ready-to-eat foods are purchased^21,32^. To address this gap, this study aimed to investigate the impact of a mass-media food safety behavior change campaign on consumer’s behavior, and on key drivers of behavior in relation to the purchase of ready-to-eat chicken in urban informal markets in Ouagadougou, Burkina Faso. Specifically, the study primarily aimed to assess associations between campaign recall and food safety purchasing behavior, and secondarily with important key behavioral determinants (i.e., intentions, knowledge, norms, attitudes and agency), and the relative importance of the campaign’s different channels in explaining differences in outcomes with campaign recall. The following research questions were addressed:

## What are the associations between

- prompted campaign recall and self-reported behavior regarding purchasing and consumption of ready-to-eat chicken at outlets?
- recall of TV, radio, billboard, or social media advertisements and self-reported behavior regarding purchasing and consumption of ready-to-eat chicken at outlets?

## What are the associations between

- prompted campaign recall and consumer intentions, knowledge, attitudes, norms, and agency regarding food safety behaviors?
- recall of TV, radio, billboard, or social media advertisements and consumer intentions, knowledge, attitudes, norms, and agency regarding food safety behaviors?

## Methods

### Study setting

The study was conducted in the capital of Burkina Faso, Ouagadougou, located in the Central Region. With a fast-growing and young population of approximately 2.4 million, population growth is twice the world average. Ouagadougou has a multiethnic and multireligious population. Development challenges include political instability, low educational attainment, lack of access to good infrastructure, water, and sanitation, and food insecurity, affecting food safety^2,33^.

### The "Bien Choisir Son Koassa!" consumer campaign

A nine-months multi-media campaign "Choose the right vendor to eat better!" ("*Bien Choisir Son Koassa Pour Mieux Manger!*") was developed with support of project partners within the larger "Pull-Push project" (See: www.ilri.org/research/projects/urban-food-markets-africa-incentivizing-food-safety-using-pull-push-approach), which aimed to reduce the burden of foodborne disease by improving the safety of fresh foods sold within urban informal markets in Burkina Faso and Ethiopia. The campaign in Burkina Faso sought to improve food safety in urban informal markets through empowering consumers who regularly purchase and consume ready-to-eat chicken in Ouagadougou to make safer choices before purchasing and consuming chicken meat at street outlets.

The campaign’s design was grounded in behavioral change theories including the Theory of Planned Behavior^34^ and Elaboration Likelihood Model^35^. Content was based on insights from prior research including chicken vendor Knowledge, Attitudes, and Practices surveys in Ouagadougou from 2021, qualitative research, and expert meetings^12,36^. Key messages targeted consumers’ behavior prior to purchasing and consuming chicken at outlets concerning: outlet and vendor hygiene; tools and hand cleanliness; using separate materials for raw chicken; ensuring high-quality chicken; handwashing; serving chicken hot; and maintaining plate cleanliness. The campaign’s creative concept was designed by Media Com, a media agency based in Ouagadougou. It focused on changing consumer knowledge, awareness, motivations and social norms surrounding food safety behaviors. Campaign messages were largely associated with positive emotions (i.e., infotainment) to stress the key benefits of choosing safe chicken, instead of focusing on risks and dangers associated with unsafe food handling. For this purpose, the campaign engaged the well-known comedian 'Moussa Petit Sergent’ as a key opinion former and influencer.

A launch ceremony on March 24, 2022 aimed to gain support from government officials, non-governmental organization, journalists, and the public, and was widely covered by local news media. From June 1, 2022 to January 9 2023, the campaign was delivered through TV and radio ads, and social media posts (i.e., Facebook, YouTube). Additionally, a project website was launched. Campaign materials are available from the project’s website (See: www.ilri.org/pull-push-project-implementing-food-safety-consumer-campaigns). Two 60-second spots featuring model consumers and a health professional providing expert food safety advice were broadcast on local and national TV stations: RTB (90 times), Savane TV (40 times), and 3TV (105 times) at different time slots during the day, in June 2022, from mid-October to mid-November 2022, and from mid-December 2022 to mid-January 2023. Moreover, a mini-series of four short video capsules featuring Moussa Petit Sergent were broadcast on 3TV and shared on YouTube and his Facebook page. Each capsule consisted of a short humorous story featuring campaign messages. A total of 180 radio spots were broadcast by local radio stations: RTB Radio, Savane FM, Omega FM, and Femina FM. More than 100 digital messages were posted on the project’s Facebook page, that had over 8000 followers with the most popular posts reaching 25,431 views. Video capsules were shared on the Facebook page of the influencer-comedian. With more than 450,000 followers, his posts reached over 1.1 million views by the end of the campaign. In June 2022, 30 billboards (4 by 3 m2) were placed throughout Ouagadougou. To widen reach and tailor messages, all content was distributed in French, *Dioula,* and *Moore*.

### Study design and sampling

To evaluate the campaign’s impact on consumer behavior and determinants, two-wave consumer panel surveys were administered before (baseline) and after the campaign (endline), with adult consumers, aged 18-49, who regularly purchased ready-to-eat chicken for immediate consumption at the market or to take-away. We aimed for a sample of 1100 consumers, anticipating a 10% attrition rate, similar to other evaluation studies and anticipating moderate (0.2) behavioral effect sizes^37,38^. Sampling used the sampling frame from previous Knowledge, Attitudes, and Practices surveys (February-June 2021) that surveyed 100 randomly-drawn outlets from a total of 622 chicken outlets in Ouagadougou^36^. From the 100 outlets, we randomly selected 25 outlets, which were localized using GPS coordinates. Enumerators stationed at selected outlets and invited every third eligible consumer purchasing chicken to participate until reaching a maximum of 44 consumers per outlet.

Inclusion criteria included: aged 18 or above; willing to join two surveys, residing permanently in Ouagadougou or within a five-kilometer radius without plans to relocate within a year, and at least twice weekly purchasing ready-to-eat chicken meat. Due to recruitment challenges, this approach shifted from every third consumer to encompassing all customers, and two additional outlets were randomly sampled to achieve the sample size, totaling 27 outlets. A total of 1063 consumers participated in baseline surveys and 943 consumers agreed to participate in a follow-up survey, resulting in an attrition rate of 11.3%. Reasons for drop-out were: no reason given (8.3%), no response (1.8%), incorrect or inactive phone number (0.9%), or unavailable (0.3%). To protect data quality, we flagged cases whose endline interviews took less than 12 min according to server metadata (*n* = 138), or when there was a failure to correctly record the start and/or end time of the interview (*n* = 62). Each case was then carefully checked to detect any inconsistencies in age, name, and occupation. A combination of short endline interview duration and inconsistencies in data led to the removal of 91 cases whose data were assessed to be less reliable, resulting in a total analytical sample of 852 consumers and a corrected attrition rate of 19.9%.

### Data collection

Surveys were pre-tested in markets in Ouagadougou as part of enumerator trainings. Questionnaires were programmed in English and French in KoboCollect on tablets. Baseline surveys were conducted pre-campaign (March 1 to 25, 2022) and endline surveys were conducted a year later, a month after the campaign’s ending (March 1-30, 2023). At baseline, ten trained enumerators (in pairs of two) interviewed consumers in-person, while awaiting their chicken order. Interviewers, with outlet owner consent, sat at a quiet corner table to avoid disturbing other consumers. At endline, six enumerators were each assigned 177 consumers to be interviewed again. Enumerators contacted consumers by phone to invite them to a follow-up interview at a central location at the market (36%), or, if not possible, via phone (64%). Interviews lasted on average 25 min and were held during outlet peak hours, starting in the late afternoon and continuing until late in the evening.

**Fig. 1:**
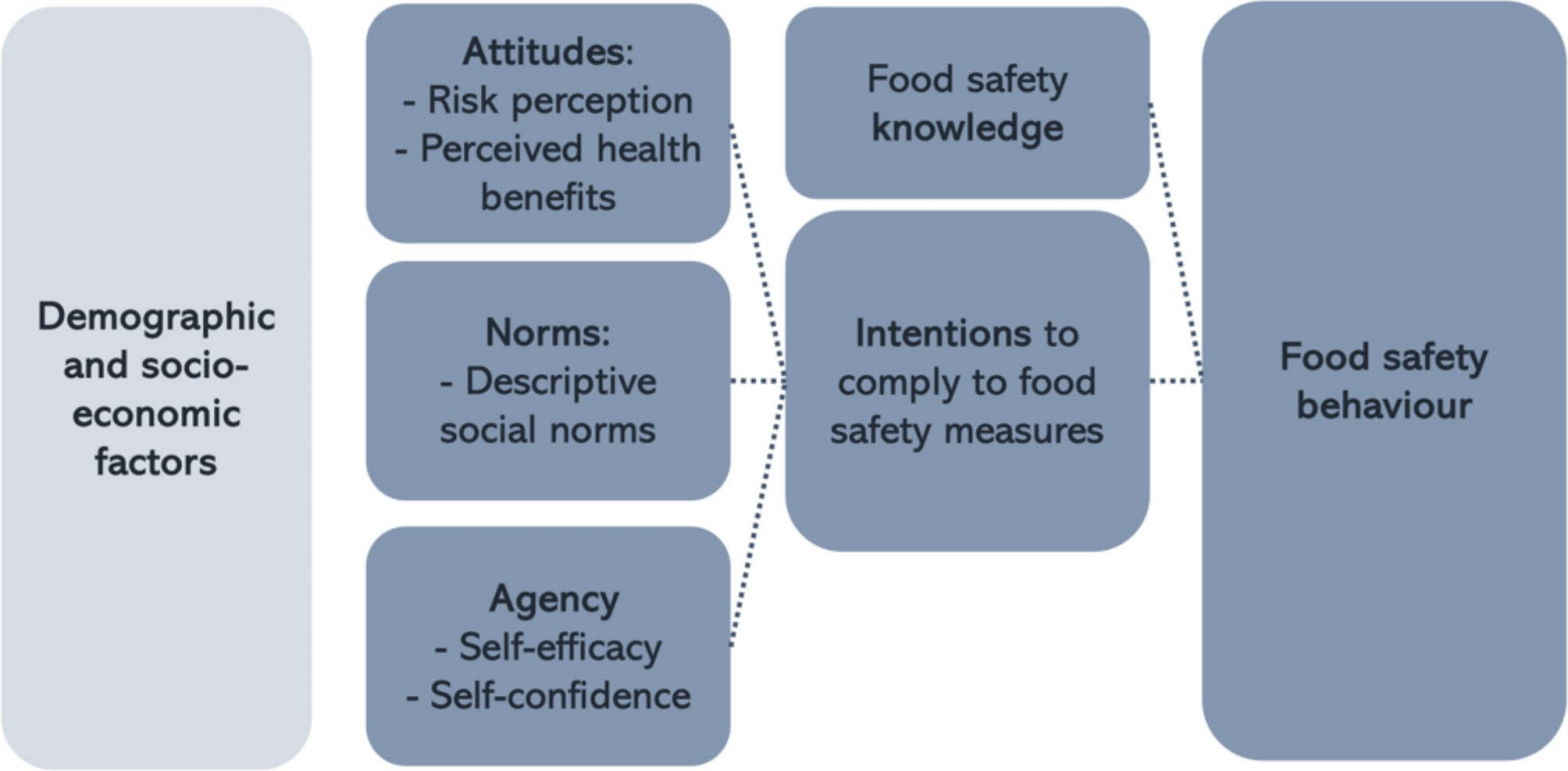
Conceptual framework - integrated behavior model. (Adapted from Montano & Kasprzyk, 2015).

Survey items were adapted from relevant food safety behavior studies due to a lack of standard questionnaires^39,43^. We aligned questions with key campaign messages and concepts of the Integrated Behavior Model. This theoretical framework is used to understand behavioral intentions and actions by considering a combination of factors that influence food safety behavior^25^. In the context of this study, we focused on intentions, knowledge, attitudes, norms, and agency (Fig. 1) related to paying attention to the cleanliness of the outlet, the vendor, and how chicken was prepared.

#### Outcome variables

The primary outcome was self-reported food safety behavior when purchasing ready-to-eat chicken, measured using eight indicators tailored to campaign key messages, scored on frequency five-point Likert scales (1 = never, 5 = always). For example, "before buying ready-to-eat chicken at an outlet: how often do you check that the outlet is visibly clean?". A total behavior score was calculated summing the eight behavior items (maximum 40 points). We also collected purchasing frequency and outlet choice indicators.

Secondary outcomes of interest included food safety knowledge, intentions, attitudes, norms and agency, highlighted dark blue in the conceptual model (Fig. 1), with survey items outlined in Table 1. Food safety knowledge was tested through seven items including four multiple-response questions on how to characterize a clean outlet, vendor hygiene, good quality chicken, and whether chicken was cooked thoroughly, and three true/false questions on handwashing, and the use of different and clean materials for raw and prepared food. For the multiple-response options, one point was assigned for each correct given answer, after which scores for these four items were rescaled to a range of 0 to 1. Other answers were coded incorrect ('0'), or correct ('1'). ‘Don’t know/not sure’ responses were assigned zero points. Summing the ten knowledge items, we created a total knowledge score (maximum score 7).

**Table 1:** Unadjusted within-subjects differences (between baseline and endline) and between-subjects difference scores (between recall and no recall groups) on outcomes of interest.

Outcome variables	Within-subject difference between baseline and endline	Between-subject differences between recall and no recall groups over time
	Mean diff, p-value	Mean diff-in-diff, p-value
Self-reported behavior *(1 = never*,* 5 = always)*		
Frequency of checking that the outlet is visibly clean	0.381, *p* < 0.001	-0.075, *p* = 0.479
Frequency of checking that the vendor is visibly clean	0.364, *p* < 0.001	-0.098. *p* = 0.347
Frequency of checking that clean materials were used for preparing food	0.217, *p* < 0.001	0.057, *p* = 0.615
Frequency of checking that vendor has used different materials for preparing raw chicken and other prepared food	0.45, *p* < 0.001	0.115, *p* = 0.335
Frequency of checking that the chicken you ordered is of good quality	0.019, *p* = 0.701	-0.071, *p* = 0.447
Frequency of washing hands with clean water and soap when eating at the outlet	0.015, *p* = 0.745	-0.006, *p* = 0.947
Frequency of checking that the chicken meat is served hot?	-0.148, *p* < 0.001	-0.115, *p* = 0.166
Frequency of checking that plates/containers are clean or washed with clean water	-0.065, *p* = 0.236	-0.022, *p* = 0.840
*Total score behavior (max. 40 points)*	*1.235*,*p < 0.001*	*-0.216*,*p = 0.722*
Intentions *(1 = never*,* 5 = always)*		
Frequency of intending to buy ready-to-eat chicken at an outlet that looks (visibly) clean	-0.077, *p* = 0.040	-0.049, *p* = 0.520
Frequency of intending to buy ready-to-eat chicken at an outlet where the vendor looks (visibly) clean	-0.054, *p* = 0.132	-0.077, *p* = 0.520
Frequency of intending to buy ready-to-eat chicken at an outlet where it is visible that separate materials are used for preparing raw chicken and other prepared food	0.067, *p* = 0.149	0.004, *p* = 0.968
Frequency of intending to buy ready-to-eat chicken at an outlet where it is visible that clean materials were used for preparing food	0.036, *p* = 0.385	0.002, *p* = 0.983
Frequency of intending to buy ready-to-eat chicken at an outlet that sells good quality chicken	-0.070, *p* = 0.028	-0.110, *p* = 0.091
Frequency of intending to wash your hands with clean water AND soap at the outlet	-0.075, *p* = 0.030	-0.077, *p* = 0.270
Frequency of intending to check if your ready-to-eat chicken is served hot at the outlet	-0.090, *p* = 0.006	-0.013, *p* = 0.847
Frequency of intending to check that ready-to-eat chicken is served on clean plates or containers that were washed with clean water	0.109, *p* = 0.005	0.109, *p* = 0.456
*Mean score intentions (max. 5)*	*-0.019 p = 0.518*	*-0.018*,*p = 0.763*
Knowledge *(max. 1 point for each correct answer)*		
What characterizes a visibly clean chicken outlet?	0.156, *p* < 0.001	0.024, *p* = 0.099
What characterizes a visibly clean vendor?	0.218, *p* < 0.001	-0.012, *p* = 0.480
It is safe when a vendor uses the same materials such as cutting boards or knives for preparing raw chicken and then other food products" (incorrect)	0.158, *p* < 0.001	-0.078, *p* = 0.062
It is safe when a vendor uses the same unwashed knife for raw chicken and other food products (incorrect)	0.183, *p* < 0.001	-0.027, *p* = 0.499
How can you tell the ready-to-eat chicken you ordered at the outlet is of good quality?	-0.023, *p* = 0.018	0.040, *p* = 0.490
When buying ready-to-eat chicken at the outlet, when should you wash your hands with water and soap?	-0.108, *p* < 0.001	-0.108, *p* < 0.001
When ready-to-eat chicken is served at the outlet, how can you tell if the chicken meat has been cooked thoroughly?	0.121, *p* < 0.001	0.008, *p* = 0.691
*Total knowledge score (max. 7)*	*0.705*,*p < 0.001*	*-0.208*,*p = 0.019*
Risk perception *(1 = very unsafe*,* 5 = very safe)*		
Extent to which consumer believes the ready-to-eat chicken consumed at the market is safe?	0.535, *p* < 0.001	0.016, *p* = 0.875
Perceived health benefits *(1 = very detrimental*,* 5 = very beneficial)*		
Consistently paying attention to food safety behaviors when buying and consuming ready-to-eat chicken is	-0.129, *p* < 0.001	-0.215, *p* < 0.001
Self-efficacy *(1 = not at all/very difficult*,* 5 = very much/very easy)*		
Confidence in consistently paying attention to food safety behaviors* when buying ready-to-eat chicken at the outlet	0.217, *p* < 0.001	0.062, *p* = 0.496
Difficulty/ease of consistently paying attention to food safety behaviours when buying ready-to-eat chicken at the outlet	0.345, *p* < 0.001	-0.018, *p* = 0.877
Perceived social (descriptive) norms *(1 = disagree*,* 5 = agree)*		
People who are important to me consistently pay attention to food safety behaviors when buying ready-to-eat chicken	0.034, *p* = 0.528	-0.135, *p* = 0.219
Access to information		
Extent to which consumer can access information required for making informed decisions regarding the consumption of ready to eat chicken at the market? (*none*,* some*,* most to all)*	-0.026, *p* = 0.316	-0.071, *p* = 0.175
Extent to which consumers feels informed about food safety of ready-to-eat chicken meat in markets? *(1 = not at all*,* 5 = a lot)*	0.205, *p* = 0.001	-0.259, *p* = 0.044
Notes: *n* = 852. * food safety behaviors defined as: "paying attention to the cleanliness of the outlet, the koassa; and tools and plates or containers; checking the quality of the chicken, and washing hands". See Supplementary Table S4 for all within-subjects differences (between baseline and endline) and between-subjects difference scores (between recall and no recall groups) on outcomes of interest		

Intentions were measured by eight items that linked each to a behavioral item, for instance: "before buying ready-to-eat chicken at an outlet: how often do you intend to check that the outlet is visibly clean?" and scored on the same Likert scale (1 = never, 5 = always). A mean intention score was calculated, as Cronbach’s alpha for scale reliability with α-values 0.90 and 0.94 indicated good internal consistency for baseline and endline intentions, respectively.

Under attitudes we assessed risk perceptions regarding the safety of chicken purchased at markets and perceived health benefits of consistently paying attention to food safety behaviors during purchasing chicken. Norms were assessed through one descriptive social norms item: "others who are important to me always pay attention to vendor food safety behaviors when purchasing chicken". Agency was assessed by two self-efficacy and self-confidence proxies. These items were all scored on five-point Likert scales (1 = very unsafe/very detrimental to my health/disagree to 5 = very safe/very beneficial for my health/agree).

#### Predictor: campaign recall

At endline, we measured unprompted and prompted recall as an indicator of whether consumers had been exposed to the campaign or not as well as which of the channels they had seen or heard. Unprompted recall (i.e., spontaneous, without prompts) was measured asking if consumers had come across any food safety message since baseline and what this message was about. This type of recall reflects 'top-of-mind’ memory while prompted recall (using visual prompts) is a better predictor of campaign-attributed recall and therefore used as predictor in this study^44^. Prompted recall was measured by asking consumers if they had seen or heard about the "Bien choisir son Koassa" campaign. Using visual prompts for in-person interviews and a fixed description of prompts if interviewed over phone, recall of each of the four campaign channels was tested (TV, radio, online, or billboard). When recalled, consumers were asked to describe the aim of the campaign; the extent to which they trusted information received (1 = not at all, 5 = a lot); and frequency of talking about the campaign with others ( 1 = never, 5 = all the time). At both timepoints, consumers were asked to what extent they perceived they had access to information about how to buy safe chicken at the market (1 = not at all, 5 = a lot).

#### Covariates

Time-variant and time-invariant demographic and socio-economic indicators at baseline included sex (F/M), age in completed years, marital status (i.e., married, never married merged with separated, divorced or widowed due to small numbers, *n* = 8), educational attainment (i.e., no [formal] education, primary, secondary, or higher than secondary education), occupation categorized into self-employed (i.e., agriculture and trade), skilled salaried, unskilled salaried, unemployed or student, head of household (female or male), household size, and number of children below 19 years old. Time-variant indicators collected at both time points included Subjective Social Status (SSS), measured by the MacArthur Scale which captures individuals’ subjective perceptions of their position in society relative to others^45^ were consumers were asked to place themselves on a 10-rung ladder based on where they feel they stand, with ten representing the highest status and one the lowest socio-economic position. Monthly food expenditure was categorized into < 50000XOF (West African CFA Franc) [85.5USD], 50001-100000XOF [171USD], and > 100.000XOF. Monthly expenditure on chicken meat was categorized into < 10000XOF [17.1USD], 10000-25000XOF [42.8USD] and > 25000XOF (1 XOF = 0.00171 USD in March 2022, 1 XOF = 0.00161 USD in March 2023). One item from the Household Hunger Scale indicated perceived household food security "in the previous nine months, was there any time that you ran out of food and was not able to buy more?" (‘yes’ coded 1)^46^. Consumers’ perceived input in decision-making regarding chicken-meat purchasing at the market (1 = no to little input, 2 = input into some decisions, 3 = input into most or all decisions) served as agency proxy. To capture recent price fluctuations^47^, we asked consumers about their perception of changes in prices of ready-to-eat chicken since baseline categorized into ‘no change’ ('0'), up to twice as high ('1'), or twice as high or more ('2'). Don’t know responses were coded as missing. One item on perceived input in decision-making with regards to food purchasing was included.

### Analyses

Data were downloaded from the KoboToolbox server and analyzed in Stata SE 16.1^48^. Descriptive and summary statistics described sample characteristics at baseline and endline, and between consumers who recalled at least one of the campaign channels (coded as 1) and did not recall (coded as 0). To detect differences over time within the total sample and between the two groups, paired t-tests and McNemar’s tests were run. Bivariate tests included paired t-tests for normally distributed continuous data and chi-square tests for categorical data to assess differences between the time-points and between recall and no recall groups. Difference scores between baseline and endline (total sample) were calculated for all items, to detect differences between the groups on scores without controlling for covariates.

We used mixed-effects linear and logistic regression models to analyze campaign recall’s association with behavior change while controlling for covariates. Mixed-effects models are well-suited for capturing the hierarchical and repeated measures nature of our dataset, allowing to account for both fixed and random sources of variability^49^. Models were fitted including prompted campaign recall and time as main fixed effects while accounting for the nested structure of the data, with consumers (level 1 units), nested within outlets (level 2 units), added as random effects. To assess associations between prompted recall and behavior and knowledge scores, linear mixed models were estimated. Residual plots showed approximately normally distributions. However, due to highly skewed data for mean intentions (ceiling effect), and a residual plot that suggested departure from the assumption of normality, we dichotomized mean intention score into low (never-often) versus high (always) intentions and fitted a binary logistic mixed model. To assess recall on risk perceptions, perceived benefits, descriptive norm, self-efficacy, and access to information, ordered logistic mixed models were estimated. Covariates included sex, age, educational attainment, and occupation measured at baseline, perceived changes in chicken prices measured at endline only, and SSS, household size, monthly expenditure on ready-to-eat chicken meat, monthly food expenditure, decision-making input, and perceived household food security measured in both surveys. Due to moderate correlation (*r* > 0.5) we removed household head, marital status, and number of children from models^50,51^. Instead of controlling for both food and chicken expenditure indicators, food expenditure data were excluded due to missing data (*n* = 50) and multicollinearity. To assess the effects of the individual channels, dummy variables were created for radio, TV, social media, and billboard recall (‘no recall’ coded 0, ‘recall’ coded 1) replacing prompted recall. As part of sensitivity analyses (available upon request), we conducted analyses with each of the five dummies separately. To address potential heteroskedasticity and improve parameter estimate reliability, we employed robust variance-covariance estimation in all models. Significance for tests were set to *p* < 0.05. As a robustness test, we also run mixed models with the 943 sample that included the ‘unreliable’ cases (data not shown). Although there were only minor differences between models in terms of outcomes associated with campaign recall, AIC and BIC values indicated poorer model fit when including the entire sample. Hence, in the remainder of this paper, we report results for the smaller sample.

### Ethics approval

Ethics approval to conduct the study was obtained from the ILRI Institutional Research Ethics Committee (IREC, number ILRI-IREC2021-63) and from the Comité d'Ethique pour la Recherche en Santé, Burkina Faso (CERS, 2020-10-220/2022-11-232). All research was performed in accordance with relevant guidelines. Participants were invited to participate in the study on a voluntary basis, written informed consent was obtained from all individual participants included in the study, and participants received a financial compensation for participating in both survey rounds.

## Results

### Sample characteristics

Table 2 presents the demographic and socio-economic characteristics of the participants at baseline and endline. The sample included 852 consumers, primarily male (85.3%), averaging 34.9 years old at baseline. About 60.5% were married. Nearly half (47.9%) were self-employed in trading, agriculture, or livestock. A third (31.9%) had skilled salaried jobs, 7.8% had unskilled jobs, and 12.4% were students or unemployed (14 respondents were unemployed). Educational attainment beyond secondary education was 44.8%, 28.8% attained up to and including secondary education, and the rest either completed primary (15.6%) or had no formal education (10.8%). Most households (93.8%) were headed by men, with an average size of 5.3 and two children under 19.

**Table 2:** Socio-demographic characteristics of consumers at baseline and endline, and differences between campaign-aware and campaign-unaware consumers at endline.

	Baseline (*n* = 852)	Endline (*n* = 852)	*p*-value	Recall (*n* = 506)	No recall (*n* = 346)	*p*-value
	n(%) or M(SD)	n(%) or (SD)		n(%) or M(SD)	n(%) or M(SD)	
Age (in completed years)	34.9 (9.8)	35.5 (9.2)	0.015	35.2 (0.4)	34.5 (0.6)	0.288
Sex						
Male	804 (85.3)			432 (85.4)	296 (85.5)	0.005
Female	139 (14.7)			74 (14.6)	50 (14.5)	
Marital status						0.083
Married	515 (60.5)			318 (64.2)	197 (56.9)	
Never married, divorced, separated or widowed	337 (39.5)			188 (35.8)	149 (43.1)	
Occupation						
Self-employed (agriculture, trading)	408 (47.9)			232 (45.8)	176 (50.9)	0.311
Skilled salaried employment	272 (31.9)			174 (34.4)	98 (28.3)	
Unskilled salaried employment	66 (7.8)			38 (7.5)	28 (8.1)	
Unemployed or student	106 (12.4)			62 (12.3)	44 (12.8)	
Educational attainment						
No (formal) education	92 (10.8)			59 (11.7)	33 (9.5)	0.608
Primary education	133 (15.6)			76 (15)	57 (16.5)	
Secondary education	245 (28.8)			140 (27.7)	105 (30.4)	
Higher than secondary education	382 (44.8)			231 (45.7)	151 (43.6)	
Household head						
Male-headed	799 (93.8)			474 (93.7)	325 (93.9)	0.594
Female-headed	53 (6.2)			32 (6.3)	21 (6.1)	
Household size	5.3 (3.5)			5.5 (3.5)	5 (3.6)	0.049
No. of children < 19 years old	2 (1.9)			2 (1.8)	1.9 (2.1)	0.242
Ran out of food and unable to purchase more						
Yes, food-insecure	41 (4.9)	70 (8.2)	0.006	42 (8.3)	30 (8.7)	0.036
No, food secure	797 (95.1)	780 (91.8)		464 (91.7)	316 (91.3)	
Subjective social status	4.8 (1.5)	4.6 (1.7)	0.003	4.7 (1.6)	4.3 (1.8)	< 0.001
Monthly food expenditure						
< 50.000XOF	243 (29.4)	123 (15.3)	< 0.001	80 (16.5)	43 (13.6)	0.522
50.001-100.000XOF	414 (50.1)	556 (69.3)		334 (68.7)	233 (70.3)	
> 100.00XOF	169 (20.5)	123 (15.3)		72 (14.8)	51 (16.1)	
Monthly chicken expenditure						
< 10.000XOF	227 (26.6)	202 (23.7)	0.072	136 (26.9)	66 (19.1)	0.025
10.000-25.000XOF	486 (57.0)	532 (62.4)		306 (60.5)	226 (65.3)	
> 25.000XOF	139 (16.3)	118 (13.9)		64 (12.7)	54 (15.6)	
Perceived change in chicken price since baseline						
No change		271 (31.8)		141 (27.9)	130 (37.6)	0.004
Up to twice as much (0-1000XOF*)		271 (31.7)		179 (35.4)	92 (26.6)	
Twice as much or more (> 1000XOF)		310 (36.4)		186 (36.8)	124 (35.8)	
Decision-making input purchasing						
Little to no input	115 (13.6)	86 (10.2)	0.001	42 (8.4)	44 (12.7)	0.118
Some input	291 (34.5)	363 (43.0)		216 (43.3)	147 (42.5)	
Input into most or all decisions	428 (51.9)	396 (46.9)		241 (48.3)	155 (44.8)	
Extent to which consumer has access to information to make informed decisions about purchasing safe ready-to-eat chicken (1 = not at all, 4 = to a high extent)						
	2.5 (1.0)	2.4 (0.8)	0.005	2.5 (0.8)	2.3 (0.8)	< 0.001

Notes: *n* = 852. missing data *n* = 8. Chi-square tests for categorical variables, t-test for continuous variables. Significance set at *p* < 0.05. *1000 XOF = 1.60895 USD.

Over time, the proportion of food-insecure households significantly increased from 4.9 to 8.2%, households spending over 50.000XOF monthly increased from 50.1 to 69.3%, and mean SSS decreased from 4.8 to 4.6. An increase was observed in the proportion of consumers who reported having “some input” into most or all decisions (34.5-43%), while the proportion of consumers with input into most/all decisions decreased (51.9-46.9%). More than two thirds of respondents perceived that chicken meant prices had at least doubled (31.7%) or more than doubled (36.4%) since baseline.

### Campaign recall

Without prompting, almost half of consumers recalled any food safety campaign since baseline (Table 3). Main campaign messages included promoting safety of chicken, improving food safety/quality (48.8%) or raise awareness on the avian influenza, chicken meat scares or scandals (i.e., dangers of genetically modified chicken, shady broilers, needles reportedly found in meat, or spoilt imported chicken). When prompted, 29.2% recalled a TV or radio (9.4%) advert, 40% had seen at least one billboard in the city, 30.6% viewed a Facebook post (29.8%), visited YouTube (1.5%) or the website (1.9%). Of those who recalled at least one campaign channel (59.4%), almost half believed the aim was to improve food safety (44.7%). Three out of four consumers trusted information received a lot or very much and almost half had at least sometimes discussed the campaign with others.

**Table 3:** Campaign recall and appraisal.

	Recall *n* (%) / mean (SD)	No recall *n* (%) / mean (SD)
Unprompted recall	424 (49.8)	428 (50.2)
Main message(s) campaign if yes:		
Message related to safety of ready-to-eat chicken	139 (32.8)	
Avian flu awareness	24 (5.7)	
Chicken meat scandals or scares	23 (5.4)	
Improving public health, WASH or nutrition	13 (3.1)	
Food safety/quality in general	207 (48.8)	
Not specified or can’t remember	18 (4.2)	
TV recall	249 (29.2)	603 (70.8)
Radio recall	80 (9.4)	772 (90.6)
Print media (poster/billboard) recall	341 (40.0)	511 (60.0)
Digital/online (Facebook, YouTube, or website) recall	261 (30.6)	591 (69.4)
Facebook recall	254 (29.8)	598 (70.2)
YouTube recall	13 (1.5)	839 (98.5)
Website recall	16 (1.9)	836 (98.1)
Prompted recall (recall of minimum one channel)	506 (59.4)	346 (40.6)
Main message(s) campaign		
Improving public health	93 (18.4)	
Improving food safety	226 (44.7)	
Improving health/nutrition knowledge	75 (14.8)	
Promoting chicken consumption	30 (5.9)	
Promoting hygiene	72 (14.2)	
Don’t know/can’t remember	10 (2.0)	
Trust in information received		
Not at all	5 (1.0)	
Somewhat	70 (13.8)	
Neutral	33 (6.5)	
A lot	79 (15.6)	
Very much	319 (63.0)	
Talked with others about campaign		
Never	117 (23.1)	
Rarely	145 (28.8)	
Sometimes	163 (32.2)	
Often	51 (10.1)	
Always	30 (5.9)	

Notes: *n* = 852.

The recall group was characterized by having a larger household size, lower food-insecurity (8.3% vs. 8.7%), higher SSS (4.7 vs. 4.3), lower monthly expenditure on chicken meat (19.1% vs. 26.9% <10000XOF). A lower proportion of respondents perceived that prices of chicken meat had not changed since baseline (27.9% vs. 37.6%).

At endline, chicken meat was less frequently purchased compared to baseline (*p* < 0.001). We observed a decrease in flamed (i.e., on firewood) chicken purchasing (66-53.1%) and an increase in consumption of braised (i.e., on charcoal) chicken (16.6-31.2%, *p* < 0.001). The average price paid for an order significantly increased from 3799.6 to 4059.4 XOF (*p* = 0.004), and consumers felt less able to access information to make informed decisions about purchasing safe chicken at the market (*p* = 0.005). No statistically significant differences were observed between those who recalled the campaign or not, except for the higher score on perceived access to information in the recall versus no recall group (*p* < 0.001) **(**Table 4).

**Table 4:** Purchasing frequency, type of dish ordered, reasons for selecting outlets between timepoints and between recall and no recall groups at endline.

	Baseline n(%)/mean(SD)	Endline	*p*	Recall	No recall	*p*
Purchasing frequency						
Less than once a week	246 (28.9)	473 (55.5)	< 0.001	272 (53.8)	201 (58.1)	0.289
Once a week	314 (36.9)	246 (28.9)		156 (30.8)	90 (26.0)	
2-3 times a week	235 (27.6)	110 (12.9)		62 (12.3)	48 (13.9)	
> 4 times a week	57 (6.7)	23 (2.7)		16 (3.7)	7 (2.0)	
Type of dish ordered						
Flamed chicken (firewood)	562 (66.0)	452 (53.1)	< 0.001	259 (51.2)	193 (55.8)	0.633
Braised chicken (charcoal)	141 (16.6)	266 (31.2)		167 (33.0)	99 (28.6)	
Fried (oil)	57 (6.7)	42 (4.9)		28 (5.5)	14 (4.1)	
Cooked with vegetables	77 (9.0)	58 (6.8)		33 (6.5)	33 (6.5)	
Roasted chicken	12 (1.4)	29 (3.3)		16 (3.2)	16 (3.2)	
Other	3 (0.4)	6 (0.7)		3 (0.6)	3 (0.6)	
Price paid	3799.6 (1288.9)	4059.4 (1965.0)	0.004	4116.7 (2498.4)	3974.6 (578.0)	0.261
Reason for selecting specific outlet (top 3 most mentioned)						
No. 1	Good taste: (66.8)	Good taste (60.2)		58.5%	62.7%	
No. 2	Processes healthy chicken (39.7)	Processes healthy chicken (48.8)		49.6%	47.7%	
No. 3	Habitual (25.1)	Loyalty/clientelism (26.2)				

Notes: *n* = 852.

**Fig. 2:**
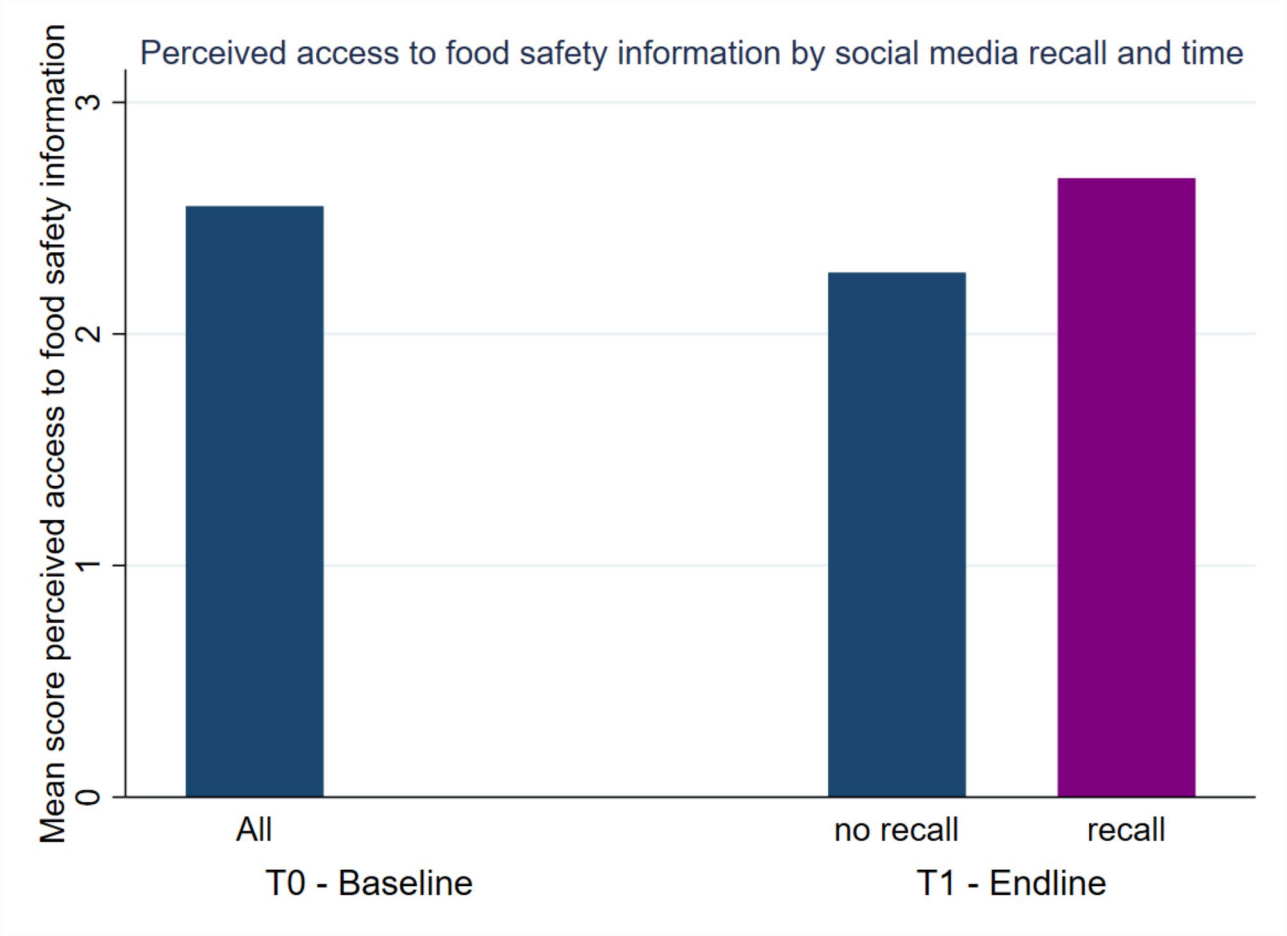
Perceived access to food safety information by recall and time.

Mixed-effects models (Table 5) estimating prompted recall on perceived access to food safety information, revealed 0.615 lower odds of higher perceived access to food safety information over time (*p* = 0.039), but those who recalled the campaign had 1.449 (*p* = 0.012) increased odds of reporting better access to food safety information compared to no recall (Fig. 2). When including dummy variables, models revealed that social media recall was associated with 1.714 (*p* = 0.006) increased odds of reporting better access to food safety information.

**Table 5:** Mixed-effects ordered logistic regression estimating associations between prompted recall (model a) and recall of specific channels (model b) and information access.

Perceived access to food safety information	a) prompted recall			b) all channels		
	aOR	p	95% CI	aOR	p	5% CI
Endline	0.615	**0.039**	0.387-0.977	0.643	0.063	0.404-1.025
Prompted recall	1.449	**0.012**	1.086-1.932			
Online recall				1.714	**0.006**	1.164-2.252
TV recall				1.319	0.141	0.912-1.906
Radio recall				0.799	0.333	0.508-1.257
Billboard recall				0.869	0.491	0.583-1.295
*RE estimate: outlet*	*0.477*		*0.309-0.736*	*0.475*		*0.305-0.739*
*RE estimate: subject*	*0.000*		*0.000-0*	*0.000*		*0.000-0*
*ICC*	*0.127*		*0.086-0.183*	*0.126*		*0.085-0.183*
*AIC*	*3738.967*			*3734.950*		
*BIC*	*3863.558*			*3875.791*		

Notes: Ref. set as baseline, or no recall. Models adjusted for: age, sex, educational attainment, occupation, perceived changes in chicken prices measured at endline, SSS, household size, monthly expenditure on ready-to-eat chicken meat, monthly food expenditure, decision-making input, and perceived household food security. Robust standard errors using vce(robust) option in STATA. Random effects (RE) estimates for outlet level and individual level. Model fit: ICC: Intraclass Correlation Coefficient; AIC: Akaike’s Information; BIC: Bayesian Information Criterion. aOR: adjusted odds ratio, SE: Standard Error.

### Self-reported behavior

Bivariate statistics (Table 1) revealed safer behavior at endline in five out of eight behavioral domains and total behavior score (*p* < 0.001), but no differences between the recall and no recall groups. Mixed-effects linear models (Table 6) confirmed that although total behavior scores were higher at endline (1.540, *p* = 0.025), no significant associations were observed with campaign recall. Since descriptive analyses showed that purchasing frequency declined over time, and to rule out potential unintended consequences of the campaign (i.e., lower purchasing frequency of chicken at the market associated with the campaign), we estimated prompted recall on purchasing frequency (see supplementary **Table** S1). Results indicated significant lower purchasing frequency of chicken meat at endline compared to baseline (aOR 0.222, *p* < 0.001), but no associations with campaign recall.

**Table 6:** Mixed-effects linear regression estimating associations between prompted recall (model a) and recall of specific channels (model b) and food safety behavior.

Total behavior score	a) prompted recall			b) all channels		
	Coef.	p	95% CI	Coef.	p	95% CI
Endline	1.338	0.070	-0.108-2.783	1.540	**0.025**	0.194-2.886
Prompted recall	0.311	0.368	-0.366-0.988			
Online recall				0.077	0.869	-0.840-0.993
TV recall				-0.295	0.591	-1.370-0.780
Radio recall				-1.048	0.136	-2.423-0.328
Billboard recall				0.346	0.461	-0.574-1.266
_constant	26.107	0.000	23.413-28.801	26.257	0.000	23.438-29.075
*RE estimate: outlet*	*3.125*		*1.779-5.492*	*3.083*		*1.771-5.369*
*RE estimate: subject*	*0.891*		*0.090-8.839*	*1.045*		*0.157-6.965*
*ICC subject*	*0.083*		*0.046-0.143*	*0.082*		*0.046-0.142*
*ICC outlet*	*0.106*		*0.057-0.189*	*0.109*		*0.061-0.188*
*AIC*	*10788.070*			*10793.480*		
BIC	10912.820			10939.920		

Notes: Ref. set as baseline, or no recall. Models adjusted for: age, sex, educational attainment, occupation, perceived changes in chicken prices measured at endline, SSS, household size, monthly expenditure on ready-to-eat chicken meat, monthly food expenditure, decision-making input, and perceived household food security. Robust standard errors using vce(robust) option in STATA. Random effects (RE) estimates for outlet level and individual level. Model fit: ICC: Intraclass Correlation Coefficient; AIC: Akaike’s Information; BIC: Bayesian Information Criterion. aOR: adjusted odds ratio, SE: Standard Error.

### Intentions

Table 1 does not reveal significant differences between the eight intention domains nor mean intention score and prompted recall. Table 7 does not show associations between time or prompted recall and mean intentions, but a significant association between TV recall and a decrease in odds (aOR 0.447, *p* = 0.001) of reporting high intentions compared to low intentions. This finding was confirmed in robustness test regressing TV recall on mean intentions separately (aOR 0.586, *p* = 0.003, data not shown).

**Table 7:** Mixed-effects linear regression estimating associations between prompted recall (models a) and recall of specific channels (models b) and food safety knowledge and intentions.

	Mean Intentions Score						Total knowledge score					
	a) prompted recall			b) all channels			a) prompted recall			b) all channels		
	**Coef**	**p**	**95% CI**	**Coef**	**p**	**95% CI**	**Coef**	**p**	**95% CI**	**Coef**	**p**	**95% CI**
Endline	0.785	0.371	0.463-1.333	0.872	0.608	0.516-1.474	0.670 (0.140)	**0.000**	0.396-0.944	0.684	**0.000**	0.411-0.975
Prompted recall	1.075	0.680	0.763-1.515	1.419	0.179	0.851-2.365	0.132 (0.060)	**0.028**	0.014-0.251			
Online recall				0.477	**0.001**	0.305-0.748				0.250	**0.000**	0.139-0.361
TV recall				0.773	0.321	0.464-1.286				0.061	0.323	-0.060-0.183
Radio recall				1.323	0.379	0.709-2.467				-0.162	0.174	-0.395-0.071
Billboard recall				0.428	0.372	-0.512-1.369				-0.041	0.622	-0.202-0.121
_constant	0.357	0.055	0.125-1.023	0.370	0.073	0.125-1.097	2.759	0.000	2.449-3.069	2.796	0.000	2.471-3.121
*RE estimate: outlet*	*0.154*		*0.061-0.388*	*0.145*		*0.056-0.370*	*0.068*		*0.042-0.110*	*0.070*		*0.043-0.116*
*RE estimate: subject*	*0.000*		*0.000-0*	*0.000*		*0.000-0*	*0.000*		*0.000-0*	*0.000*		*0.000-0*
*ICC*	*0.045*		*0.018-0.105*	*0.042*		*0.017-0.101*	*0.091*		*0.060-0.135*	*0.095*		*0.062-0.143*
*AIC*	*1451.308*			*1445.952*			*4200.564*			*4194.179*		
*BIC*	*1565.203*			*1576.117*			*4325.306*			*4335.192*		

Notes: Ref. set as baseline, or no recall. Models adjusted for: age, sex, educational attainment, occupation, perceived changes in chicken prices measured at endline, SSS, household size, monthly expenditure on ready-to-eat chicken meat, monthly food expenditure, decision-making input, and perceived household food security. Robust standard errors using vce(robust) option in STATA. Random effects (RE) estimates for outlet level and individual level. Model fit: ICC: Intraclass Correlation Coefficient; AIC: Akaike’s Information; BIC: Bayesian Information Criterion. aOR: adjusted odds ratio, SE: Standard Error.

### Knowledge

**Fig. 3:**
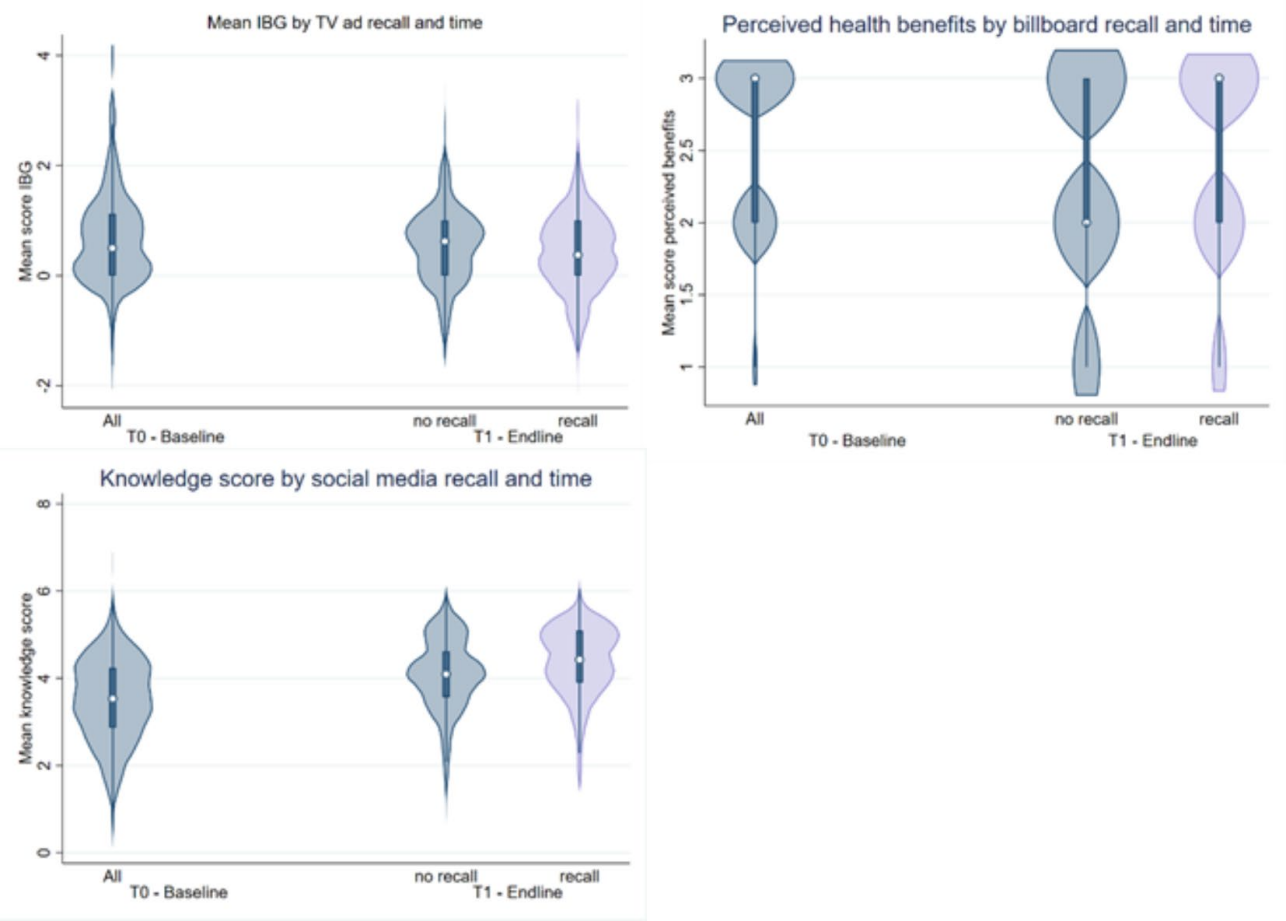
Knowledge, perceived health benefits, and IBG scores with recall and time.

At endline and among consumers who recalled the campaign, total knowledge scores were significantly higher (*p* < 0.001, Table 1). Mixed-effects models (0.670, 0.684, p = < 0.001), see Table 7, confirmed the significantly higher knowledge score at endline compared to baseline (Fig. 3). The recall group had a significantly higher knowledge score then the group who did not recall the campaign (0.132, *p* = 0.028). Social media recall was associated with a 0.250 unit increase in total knowledge score (*p* < 0.001).

### Attitudes

Consumers’ perception that ready-to-eat chicken at an outlet is safe to eat, was significantly higher at endline, after the campaign (*p* < 0.001, Table 1). Mixed-effects models confirmed that the odds of reporting higher risk perceptions, were higher at endline (Table 8, aOR 3.008 and aOR 2.832, *p* < 0.001, Fig. 2), but no association was found with campaign recall. Bivariate statistics (Table 1) indicated that consumers’ perceived health benefits of consistently paying attention to food safety behaviors when purchasing ready-to-eat chicken at the market were lower at endline and with recall (*p* < 0.001). Mixed-effects models (aOR 0.589, *p* = 0.006, and aOR 0.680, *p* = 0.046) confirmed that at endline, consumers were less likely to perceive paying attention to food safety behavior as beneficial to their health. However, increased odds (aOR 2.046, *p* = 0.006) of higher perceived health benefits were observed among consumers who recalled having seen a billboard in the city (Fig. 3). Additionally, a significant association between TV ad recall and lower perceived health benefits (aOR 0.440, p = < 0.001) was observed and confirmed in robustness tests.

**Table 8:** Mixed-effects ordered logistic regression estimating associations between prompted recall (models a) and recall of specific channels (models b) and food safety risk perceptions, perceived benefits, descriptive norms, self-efficacy and perceived access to information.

Risk perception (not safe to very safe)	a) prompted recall			b) all channels		
	aOR	p	95% CI	aOR	p	95% CI
Endline	3.008	**0.000**	2.051 (4.413)	2.832 (0.536)	**0.000**	1.955-4.103
Prompted recall	0.866	0.360	0.637 (1.178)			
Online recall				1.235 (0.299)	0.383	0.769-1.983
TV recall				1.314 (0.220)	0.104	0.945-1.825
Radio recall				0.875 (0.204)	0.568	0.554-1.383
Billboard recall				0.663	0.124	0.392-1.119
*RE estimate: outlet*	*1.453*		*0.765*	*1.453*		*0.763-2.765*
*RE estimate: subject*	*0.696*		*0.265*	*0.689*		*0.268-1.768*
*ICC subject*	*0.267*		*0.164*	*0.267*		*0.164-0.405*
*ICC outlet*	*0.395*		*0.265 (*	*0.394*		*0.265-0.541*
*AIC*	*4852.788*			*4848.089*		
*BIC*	*4988.377*			*4848.089*		
Perceived benefits food safety behavior (low to high)	**a) prompted recall**			**b) all channels**		
Endline	0.589	**0.006**	0.403-0.861	0.680	**0.046**	0.465-0.994
Prompted recall	1.248	0.143	0.928-1.678			
Online recall				0.916	0.716	0.570-1.472
TV recall				0.440	**0.000**	0.336-0.578
Radio recall				0.779	0.242	0.514-1.183
Billboard recall				2.046	**0.006**	1.233-3.394
*RE estimate: outlet*	*0.210*		*0.103-0.425*	*0.216*		*0.106-0.438*
*RE estimate: subject*	*0.000*		*0.000-0*	*0.000*		*0.000-0*
*ICC*	*0.060*		*0.060 − 0.020*	*0.062*		*0.031-0.118*
*AIC*	*2602.671*			*2584.893*		
*BIC*	*2721.990*			*2720.482*		
Descriptives norms (disagree to disagree)	**a) prompted recall**			**b) all channels**		
Endline	0.984	0.944	0.634-1.529	1.089	0.692	0.715-1.659
Prompted recall	1.131	0.135	0.962-1.330			
Online recall				0.989	0.941	0.737-1.327
TV recall				1.133	0.543	0.758-1.693
Radio recall				0.963	0.857	0.643-1.444
Billboard recall				0.865	0.367	0.631-1.186
*RE estimate: outlet*	*0.415*		*0.195-0.884*	*0.412*		*0.188-0.902*
*RE estimate: subject*	*0.000*		*0.000-0*	*0.000*		*0.000-0*
*ICC*	*0.112*		*0.056-0.212*	*0.111*		*0.054-0.215*
*AIC*	*4814.994*			*4820.680*		
*BIC*	*4945.160*			*4967.117*		
Self-efficacy: Confidence in consistently paying attention to food safety behaviors when buying ready-to-eat chicken at the outlet	**a) prompted recall**			**b) all channels**		
Endline	1.501	**0.045**	1.009-2.232	1.409 (0.267)	0.070	0.972-2.043
Prompted recall	0.959	0.744	0.746-1.233			
Online recall				1.084 (0.163)	0.590	0.808-1.456
TV recall				0.738 (0.108)	**0.038**	0.554--.983
Radio recall				1.016 (0.207)	0.938	0.681-1.515
Billboard recall				1.285 (0.291)	0.269	0.824-2.004
*RE estimate: outlet*	*0.252*		*0.125-0.506*	*0.255 (0.091)*		*0.127-0.514*
*RE estimate: subject*	*0.107*		*0.004-2.883*	*0.108 (0.184)*		*0.004-3.037*
*ICC subject*	*0.069*		*0.034-1.134*	*0.070 (0.024)*		*0.035-0.136*
*ICC outlet*	*0.098*		*0.040-0.221*	*0.100 (0.045)*		*0.040-0.227*
*AIC*	*3875.104*			*3872.852*		
*BIC*	*4010.693*			*4013.865*		
Self-efficacy: Difficulty/ease of consistently paying attention to food safety behaviours when buying ready-to-eat chicken at outlet	**a) prompted recall**			**b) all channels**		
Endline	1.636 (0.279)	**0.004**	1.171-2.285	1.624	0.**005**	1.155-2.283
Prompted recall	1.014 (0.140)	0.922	0.773-1.329			
Online recall				1.211	0.201	0.903-1.625
TV recall				0.907	0.561	0.652-1.261
Radio recall				1.166	0.422	0.802-1.697
Billboard recall				0.929	0.687	0.648-1.331
*RE estimate: outlet*	*0.051*		*0.016-0.163*	*0.053*		*0.017-0.162*
*RE estimate: subject*	*0.062*		*0.002-2.329*	*0.060*		*0.001-2.532*
*ICC subject*	*0.015*		*0.005-0.046*	*0.015*		*0.005-0.046*
*ICC outlet*	*0.033*		*0.004-0.217*	*0.033*		*0.004-0.212*
*AIC*	*5049.499*			*5048.871*		
*BIC*	*5185.088*			*5189.884*		

Notes: Ref. set as baseline, or no recall. Models adjusted for: age, sex, educational attainment, occupation, perceived changes in chicken prices measured at endline, SSS, household size, monthly expenditure on ready-to-eat chicken meat, monthly food expenditure, decision-making input, and perceived household food security. Robust standard errors using vce(robust) option in STATA. Random effects (RE) estimates for outlet level and individual level. Model fit: ICC: Intraclass Correlation Coefficient; AIC: Akaike’s Information; BIC: Bayesian Information Criterion. aOR: adjusted odds ratio, SE: Standard Error.

### Norms

No significant associations were found between time or campaign recall and the extent of agreeing to social descriptive norms (Table 8).

### Agency

Self-efficacy, consumers’ confidence in their abilities to adhere to food safety behaviors, appeared to be significantly higher at endline but no differences were observed between consumers who recalled and who did not recall the campaign (Table 1). Mixed-effects models (Table 8) confirmed significant positive associations between time and higher confidence in consistently paying attention to food safety behaviors when purchasing chicken (aOR 1.501, *p* = 0.045, and aOR 1.636, *p* = 0.004), but not with campaign recall. While TV recall seemed to be negatively associated with the odds of reporting higher self-efficacy (aOR 1.35, *p* = 0.038), this association disappeared in a robustness analysis regressing TV recall only on self-efficacy.

### Additional analysis: the Intention-Behavior Gap

Considering relatively high intentions at baseline, but non-significant changes in self-reported behavior, we modelled the association between campaign-recall and intention-behavior gap (IBG). The IBG represents the disconnect between intending to practice safe food habits and actually implementing behaviors. A larger gap indicates poorer progress towards behavior change^52^. IBG was calculated for all eight behavior domains by subtracting intention scores from self-reported behavior scores. Next, means IBG scores (range 1-5) were calculated: scores approaching five represented a large IBG and scores towards 1 indicated a smaller gap. Linear mixed-effects models, provided as Supplementary Information (**Table** S2 and S3), showed a general decrease in IBG over time (-0.172, *p* = 0.002) and a significant 0.159 unit reduction in IBG (*p* = 0.035) with TV recall indicating a stronger alignment between what a consumer intended to do and actual (reported) behavior (Fig. 3).

## Discussion

This study evaluated the impact of a mass-media communication campaign on food safety behavior and drivers of behavior including traditional media channels alongside social media, to ultimately enhance the safety of ready-to-eat chicken sold in urban informal markets in Ouagadougou, Burkina Faso. The study advances the literature on the effects of consumer-focused food safety interventions in Sub-Saharan Africa with a specific focus on buying and consuming ready-to-eat chicken sold in informal markets, as a potential strategy for increasing demand for safer food, ultimately leading to improved vendor practices and reduced foodborne disease.

Over half of consumers recalled the campaign. Social media, TV ads, and billboards were recalled by a third of the panel, while radio ads were only remembered by one in ten consumers. In line with findings from other food safety campaigns including a food safety campaign in Ethiopia^22^, campaign recall, especially through social media, linked to better perceived access to food safety information and higher knowledge scores. This suggests that the campaign’s infotainment approach was highly effective^28^. Moreover, billboard recall was associated with a two-fold increase in the odds of reporting higher perceived health benefits of paying attention to food safety behaviors when purchasing ready-to-eat chicken.

In both high- and low-income contexts including Sub-Saharan Africa, mass-media campaigns have effectively influenced various health behaviors^53^, including HIV/AIDS prevention^54^, nutrition^55^, and food safety^21^. In this study, we found no associations between campaign-recall and self-reported behavior. Translating knowledge and intentions into action, as seen in public health and food safety promotion, remains a common challenge^56^. Despite the nine-months campaign, which is considered long, effects might still have been constrained, given the time needed for behavior change. For instance, a meta-analysis of the effects of similar health communication campaigns in the United States only showed a modest effect size of 5% on behavior^57^. However, in our study, we found that behavior notably improved over time regardless of campaign recall. Recall bias with participants incorrectly recalling if they had been exposed to the campaign, might have caused a failure to detect significant associations. Alternatively, the survey’s repeated interview approach might have boosted participants’ measured food safety awareness and behavior, for all participant groups, independent of campaign recall or external factors influencing behavior. Although the campaign did raise awareness, sustained behavior change requires repeated exposure waves and ongoing investments^20^, alongside interpersonal interventions such as household extension worker visits^58^.

The campaign’s use of social media and a prominent national comedian as an influencer, going beyond traditional TV and radio ads, was successful. With an increasing number of consumers owning smartphones with internet access, especially in our panel (adult men, age, and those from wealthier households that typically frequent chicken restaurants in Ouagadougou) influencers or celebrities can effectively target behavior of their followers through posting online content or calls to actions, tapping into emotions for greater impact^59^. This study’s findings on improved food safety knowledge through social media recall are especially promising and contribute to the scarce literature on social media’s role in food safety interventions, particularly in low-and middle-income countries^60^. There is mixed evidence on the impact of food safety media campaigns relying on social media. For instance, a social media campaign showed no impact in the prevention of Lassa Fever in Nigeria^61^. A review on social media use in food safety campaigns underscored the importance of traditional media channels^60^. However, in China, the use of social media was positively related to cognitive, emotional, and behavioral responses to food safety concerns^62^. On the condition that followers trust online sources and correct information is provided, influencers may act as role model, creating awareness and changing norms that ultimately help consumers to recognize and prevent unsafe behavior^20^.

Many consumers already had high intentions to perform food safety behaviors at baseline, which is encouraging as a key driver of change, but it could also indicate that consumers face constraints to translate their intentions into actual behaviors due to non-behavioral and contextual factors responsible for consumers’ capability to buy safer chicken. Examples in this context include safe chicken availability, purchasing power, social influences, or habits^63,64^. Moreover, food safety knowledge and behavior could differ between sociodemographic groups or with educational attainment^65^. Although the focus of our study was on evaluating the potential effects of campaign-recall on food safety behavior, adjusted models consistently linked socio-economic status and recent food price changes to altered behavior, potentially reducing consumers’ intentions to purchase safe chicken. While it is plausible that the campaign increased consumer awareness of poor food safety practices with this leading to consumers choosing to avoid purchasing chicken meat due to perceived safety concerns, extra analyses (not shown) indicated that the reduced chicken purchasing post-campaign was unrelated to campaign recall. Reduced purchasing might be due to recent political and health crises such as the COVID-19 pandemic which elevated living costs and impacted food access and availability. Price instabilities and lower purchasing power may have led consumers to demand or be more willing to pay for cheaper, less nutritious, and possibly less safe food^66,67^. To support consumers to choose safer food, good availability and affordability of safe food in these markets is crucial, alongside awareness on the significant costs placed on household incomes due to foodborne disease^68,69^. Future research into the effects of socio-economic status, consumer willingness to pay for safer ready-to-eat chicken meat, as well as the important role of contextual factors could provide more information into potential effects of economic influences.

Behavioral intentions tend to remain relatively consistent over time and are often less responsive to campaigns aimed at altering them, or more likely to influence information-processing related behavior changes^70^. Previous research showed that behavioral intentions do not always predict actual behavior, underscoring the complex nature of the relationship between intention and behavior. For example, intentions have been shown to only account for 18 to 23% of the variance in several health-related behaviors^71^. The larger the gap between intention and behavior, the more likely behavior deviates from intentions^52^. Our study found no significant links between reported intentions, behavior and campaign recall. However, among those recalling TV ads, the intention-behavior gap reduced. While minimizing this gap is crucial in promoting behavior change as better alignment implies a higher likelihood of adherence to food safety practices, the stronger consistency between intentions and behavior could also be a result of a reduction in intentions to buy safer food, due to above-mentioned factors, rather than indicating an improvement in food safety behavior.

Theories on optimistic bias posit that when consumers feel that they are immune to foodborne illness or food hazards, they are less likely to respond to health advice and thus report lower intentions^65^. For effective campaigns and behavior change, consumers must perceive risks in buying unsafe ready-to-eat chicken and its associated costs. Weaker intentions to pay attention to vendor food safety behavior might also relate to declining risk perceptions over time (e.g. believing that market-purchased chicken is generally safe)^72^.

TV recall was associated with reduced perceived health benefits and intentions for safe food behavior. The extensive information from TV ads as opposed to the concise social media messages, might lead to consumers struggling to remember key campaign messages, possibly explaining these findings. Discrepancies between visual and audio content in TV spots, where viewers see but do not hear the message, might have affected audience perception and response. As visual learning is considered important^73^, perceived mismatches between audio and visuals could confuse viewers, leading to content disregard^35^. In this campaign, TV ads included health expert advising on food safety behaviors as opposed to the humorous and positive tone in the other campaign channels. Consumer disregard for expert food safety assessments may have created an 'expert-lay discrepancy.' Information-based campaigns alone might therefore not effectively shift social norms^74^.

### Methodological considerations

This study’s repeated-measures design provided unique insights into the associations between campaign recall, self-reported behaviors, and behavioral determinants. With a representative panel and high follow-up rates, it significantly adds to the otherwise limited data on consumer-focused interventions in an sub-Saharan African context^75^. While we used a theory-based approach guided by the Integrated Behavior Model to assess effects of campaign recall on important behavioral drivers, we recognize that unmeasured contextual constraints or prevailing social and cultural beliefs could affect these associations. Exploring psychical and social contextual factors might be beneficial for developing future interventions, such as market environments and infrastructure, food availability and accessibility, or political climate, or socio-cultural beliefs surrounding food safety that might influence behavior. The absence of a contemporaneous control group poses a challenge in attributing the observed effects solely to the campaign, but parallel intervention control arms in a trial design was not possible considering the campaign could not be limited to the intervention group, but was broadcasted to the plarger population. By employing diverse question formats, the study minimized the impact of social-desirability bias in self-reported behavior^76^. However, some discrepancies between self-reported and observed behaviors have been observed in similar research^77,78^. Future investigations could include observations of consumer and vendor practices alike to diminish the risks of overestimating self-reported behavior^56^. To mitigate the potential for recall bias, we used visual prompts during interviews, and a verbal description of visuals instead during phone interviews. Nevertheless, recall challenges during phone interviews, may potentially have led to underreporting, consequently affecting the models. Hence, robust cluster randomized-controlled trials could potentially provide more insights into better understanding whether consumers had been exposed to the campaign as well as explore consumer-vendor interactions. Adding qualitative methods could reveal in-depth insights into food culture and consumer motives, including willingness to pay for safer food. Moreover, understanding the interrelations and pathways between the behavioral determinants and behavior would provide further insights to inform the design of effective food safety interventions that engage consumers. For this, standardized indicators for measuring food safety behavioral determinants are essential.

## Conclusion

This study offers novel insights in the potential impact of a consumer-focused food safety intervention in urban informal markets in Burkina Faso, through providing a comprehensive and theory-based analysis of consumer food safety attitudes and practices when purchasing and consuming chicken meat at street outlets. Campaign recall revealed a 1.7-fold increase in consumers reporting improved access to food safety information, heightened knowledge, and two-fold increases in odds of higher perceived benefits in consistently practicing safe food behaviors while purchasing chicken at these outlets. The inclusion of social media influencers alongside traditional media channels such as TV, radio ads, and billboards could be a particularly valuable and effective strategy in raising awareness and knowledge in this context. For enhancing consumer food safety behavior, practices should focus not only on generating demand and increasing knowledge and awareness, but also on improving consumer confidence in purchasing and consuming safe food at market outlets. Practices could for instance include establishing quality criteria or food labeling that consumers may use to assess the safety of chicken meat sold at outlets, buying from certified vendors, or leveraging branding strategies^79^. However, to guide future efforts to enhance food safety on a larger scale, investments are needed in meaningful and tailored interventions that will transform market regulations and infrastructure to enable the sale of safe ready-to-eat food. Finally, in order for consumer demand for safer chicken meat and hygienic food handling at street restaurants to serve as a ‘pull’ strategy to drive vendors in Ouagadougou to improve their practices, vendors should be incentivized, equipped, and trained to supply safer food at the market through for example ongoing participatory training or peer-to-peer approaches^80^.

## Electronic supplementary material

Below is the link to the electronic supplementary material.

Supplementary Material 1

## Data Availability

The datasets generated and analysed during the current study are available from the International Livestock Research Institute’s ILRI DataPortal, via https://hdl.handle.net/20.500.11766.1/FK2/QYJHUC.
