# Supplementary material for: Empowering consumers to purchase safe ready-to-eat chicken from street restaurants in Ouagadougou, Burkina Faso: impact of a multi-media behavior change campaign
Source: Sci Rep. 2024 Oct 21;14:24718. doi: 10.1038/s41598-024-76123-4 (PMC11494104; doi:10.1038/s41598-024-76123-4)
Supplement: Supplementary file 1 — Supplementary Material 1 [file 41598_2024_76123_MOESM1_ESM.pdf]

# Supplementary Information

**Title**

Empowering consumers to purchase safe ready-to-eat chicken from street restaurants in Ouagadougou, Burkina Faso: Impact of a multi-media behavior change campaign

**Author names and affiliations:**

Donya S. Madjdian[a]*, Marcel van Asseldonk[b], Elise F. Talsma[c], Michel Dione[d], Guy Ilboudo[d], Kristina Roesel[e], Delia Grace[e,f], Theodore J.D. Knight-Jones[e], Emely de Vet[a,g]

[a]Consumption and Healthy Lifestyles, Wageningen University & Research, Wageningen, the Netherlands.

[b]Wageningen Economic Research, Wageningen, the Netherlands

[c]Division of Human Nutrition & Health, Wageningen University & Research, the Netherlands

[d]International Livestock Research Institute (ILRI), Ouagadougou, Burkina Faso

[e]International Livestock Research Institute (ILRI), Nairobi, Kenya

[f]Natural Resources Institute, University of Greenwich, Chatham, United Kingdom

[g]University College Tilburg, Tilburg University, Tilburg, The Netherlands

***Corresponding author:** donya.madjdian@wur.nl

**Table S1.** Mixed-effects ordered logistic regression on the association between campaign recall and purchasing frequency

| **Mixed-effects logistic regression** | **Model Sa. purchasing frequency recall any media channel** | | | | | **Model Sb. purchasing frequency- recall specific channels** | | | | |
|---|---|---|---|---|---|---|---|---|---|---|
| | **aOR** | **SE** | **p** | **95%CI** | | **aOR** | **SE** | **p** | **95% CI** | |
| **Endline** | 0.222 | 0.045 | 0.000 | 0.149 | 0.331 | 0.260 | 0.048 | 0.000 | 0.181 | 0.375 |
| **Prompted recall** | 1.359 | 0.225 | 0.064 | 0.982 | 1.881 | | | | | |
| **Online recall** | | | | | | 1.030 | 0.222 | 0.891 | 0.676 | 1.570 |
| **TV recall** | | | | | | 0.719 | 0.157 | 0.131 | 0.469 | 1.103 |
| **Radio recall** | | | | | | 0.770 | 0.200 | 0.313 | 0.462 | 1.281 |
| **Billboard recall** | | | | | | 1.385 | 0.232 | 0.052 | 0.997 | 1.924 |
| RE estimate: outlet & consumer | 0.384 | 0.210 | | 0.132 | 1.123 | 0.388 | 0.209 | | 0.135 | 1.117 |
| ICC subject | 0.105 | 0.051 | | 0.105 | 0.051 | 0.105 | 0.051 | | 0.039 | 0.254 |
| AIC | 3246.139 | | | | | 3250.736 | | | | |
| BIC | 3370.881 | | | | | 3391.749 | | | | |

Notes: Ref. set as baseline, or no recall. Models adjusted for: age, sex, educational attainment, occupation, perceived changes in chicken prices measured at endline, SSS, household size, monthly expenditure on ready-to-eat chicken meat, monthly food expenditure, decision-making input, and perceived household food security. Robust standard errors using vce(robust) option in STATA. Random effects (RE) estimates for outlet level and individual level. Model fit: ICC: Intraclass Correlation Coefficient; AIC: Akaike's Information; BIC: Bayesian Information Criterion. aOR: adjusted odds ratio, SE: Standard Error.

**Table S2.** Mixed effects linear regression on the association between campaign recall and mean Intention-Behavior Gap

| | **Model 10a. Mean IBG** | | | | | **Model 10b. Mean IBG** | | | | |
|---|---|---|---|---|---|---|---|---|---|---|
| | **Coef.** | **SE** | **p** | | **95% CI** | **Coef.** | **SE** | **p** | | **95% CI** |
| **Endline** | -0.172 | 0.054 | 0.002 | -0.278 | -0.066 | -0.159 | 0.049 | 0.001 | -0.256 | -0.063 |
| **Prompted recall** | -0.022 | 0.048 | 0.645 | -0.117 | 0.073 | | | | | |
| **Online recall** | | | | | | 0.056 | 0.067 | 0.399 | -0.074 | 0.187 |
| **TV recall** | | | | | | -0.144 | 0.068 | 0.035 | -0.278 | -0.010 |
| **Radio recall** | | | | | | -0.037 | 0.083 | 0.655 | -0.199 | 0.125 |
| **Billboard recall** | | | | | | 0.003 | 0.054 | 0.960 | -0.103 | 0.108 |
| **_constant** | 0.759 | 0.179 | 0.000 | 0.408 | 1.109 | 0.768 | 0.181 | 0.000 | 0.414 | 1.122 |
| RE estimate: outlet | 0.034 | 0.014 | | 0.148 | 0.079 | 0.035 | 0.015 | | 0.015 | 0.082 |
| RE estimate: subject | 0.059 | 0.021 | | 0.029 | 0.119 | 0.059 | 0.211 | | 0.029 | 0.119 |
| ICC subject | 0.061 | 0.023 | | 0.029 | 0.124 | 0.063 | 0.024 | | 0.029 | 0.129 |
| ICC outlet | 0.165 | 0.035 | | 0.108 | 0.245 | 0.168 | 0.034 | | 0.110 | 0.246 |
| AIC | 3757.098 | | | | | 3756.324 | | | | |
| BIC | 3881.840 | | | | | 3897.337 | | | | |

Notes: Ref. set as baseline, or no recall. Models adjusted for: age, sex, educational attainment, occupation, perceived changes in chicken prices measured at endline, SSS, household size, monthly expenditure on ready-to-eat chicken meat, monthly food expenditure, decision-making input, and perceived household food security. Robust standard errors using vce(robust) option in STATA.

Random effects (RE) estimates for outlet level and individual level. Model fit: ICC: Intraclass Correlation Coefficient; AIC: Akaike's Information; BIC: Bayesian Information Criterion. aOR: adjusted odds ratio, SE: Standard Error.

**Table S3.** Intention-Behavior gaps for each behavioral domain

| Intention | Behavior | Gap at t0 | Gap at t1 | Diff of gap between base- and endline | Diff gaps between aware and non-aware at t1 |
|---|---|---|---|---|---|
| How often do you intend to buy ready-to-eat chicken at an outlet that looks (visibly) clean? | How often do you check that the outlet is visibly clean? | 0.81 (1.30), p<0.001 | 0.37, p<0.001 | -0.44, p<0.001 | 0.09, p=0.158 |
| How often do you intend to buy ready-to-eat chicken at an outlet where the vendor looks (visibly) clean? | How often do you check that the vendor is visibly clean? | 0.81 (0.04), p<0.001 | 0.24 (0.03), p<0.001 | -0.40, p<0.001 | 0.12, p=0.059 |
| How often do you intend to buy ready-to-eat chicken at an outlet where you can see that separate materials are used for preparing raw chicken and other prepared food? | How often do you check that the vendor has used different materials for preparing raw chicken and other prepared food? | 1.27 (1.45), p<0.001 | 0.89 (1.27), p<0.001 | -0.38, p<0.001 | -0.04, p=0.663 |
| How often do you intend to buy ready-to-eat chicken at an outlet where you can see that clean materials were used for preparing food? | How often do you check that clean materials were used for preparing food? | 0.95 (0.05), p<0.001 | 0.75 (0.04), p<0.001 | -0.20, p<0.001 | 0.06, p=0.400 |
| How often do you intend to buy ready-to-eat chicken at an outlet that sells good quality chicken? | How often do you check that the chicken you ordered is of good quality ? | 0.55 (0.04), p<0.001 | 0.48, p<0.001 | -0.07, p=0.089 | 0.07, p=0.256 |
| How often do you intend to wash your hands with clean water AND soap at the outlet? | How often do you wash your hands with clean water AND soap at the outlet? | 0.38 (0.03), p<0.001 | 0.31 (0.03), p<0.001 | -0.07, p=0.087 | 0.01, p=0.803 |
| How often do you intend to check if your ready-to-eat chicken is served hot at the outlet? | How often do you check that the chicken meat is served hot? | 0.21 (0.03), p<0.001 | 0.29 (0.03), p<0.001 | 0.08, p=0.039 | **0.11, p=0.042** |
| How often do you intend to check that your ready-to-eat chicken is served on clean plates or containers that have been washed with clean water at the outlet? | How often do you check that plates/containers are clean or washed with clean water? | 0.62, p<0.001 | 0.78, p<0.001 | 0.15, p=0.004 | 0.07, p=0.367 |

**Table S4.** Extended version of Table 4: adjusted within-subjects differences (between baseline and endline) and between-subjects difference scores (between recall and no recall groups) on outcomes of interest Table 4 extended

| Outcome variables | Baseline (t0) | | | Endline (t1) | | | Δ t1-t0 (within-subject) | Difference-in-difference: Δt1-t0 between campaign-aware and unaware (between-subjects) |
|---|---|---|---|---|---|---|---|---|
| | **All (n=852)** | **Unaware (n=347)** | **Aware (n=505)** | **All (n=852)** | **Unaware (n=347)** | **Aware (n=505)** | **All (n=852)** | **All (n=852)** |
| | Mean (SD) | | | | | | Diff, p-value | Diff-in-diff p-value |
| **Self-reported behavior** | | | | | | | | |

| | | | | | | | | |
|---|---|---|---|---|---|---|---|---|
| How often do you check that the outlet is visibly clean? *(1=never, 5=always)* | 3.80 (1.33) | 3.74 (1.38) | 3.84 (1.30) | 4.18 (0.95) | 4.08 (0.99) | 4.26 (0.90) | 0.381, p<0.001 | -0.075, p=0.479 |
| How often do you check that the vendor is visibly clean? *(1=never, 5=always)* | 3.98 (1.25) | 3.94 (1.29) | 4.00 (1.22) | 4.34 (0.91) | 4.24 (0.96) | 4.40 (0.88) | 0.364, p<0.001 | -0.098. p=0.347 |
| When buying ready-to-eat chicken at an outlet: how often do you check that clean materials were used for preparing food? *(1=never, 1=always)* | 3.50 (1.36) | 3.40 (1.42) | 3.57 (1.32) | 3.72 (0.99) | 3.65 (0.98) | 3.76 (0.99) | 0.217, p<0.001 | 0.057, p=0.615 |
| How often do you check that the vendor has used different materials for preparing raw chicken and other prepared food? *(1=never, 5=always)* | 3.06 (1.43) | 2.99 (1.45) | 3.11 (1.41) | 3.48 (1.01) | 3.50 (1.00) | 3.52 (1.05) | 0.45, p<0.001 | 0.115, p=0.335 |
| How often do you check that the chicken you ordered is of good quality ? *(1=never, 5=always)* | 4.18 (1.12) | 4.14 (1.14) | 4.21 (1.11) | 4.20 (0.96) | 4.12 (0.99) | 4.26 (0.94) | 0.019, p=0.701 | -0.071, p=0.447 |
| How often do you wash your hands with clean water and soap at the outlet? *(1=never, 5=always)* | 4.33 (1.07) | 4.29 (1.08) | 4.36 (1.06) | 4.35 (0.90) | 4.30 (0.90) | 4.38 (0.85) | 0.015, p=0.745 | -0.006, p=0.947 |
| How often do you check that the chicken meat is served hot? *(1=never, 5=always)* | 4.50 (0.89) | 4.48 (0.89) | 4.52 (0.88) | 4.36 (0.89) | 4.18 (0.95) | 4.42 (0.86) | -0.148, p<0.001 | -0.115, p=0.166 |
| How often do you check that plates/containers are clean or washed with clean water? *(1=never, 5=always)* | 3.87 (1.26) | 3.80 (1.27) | 3.92 (1.25) | 3.81 (1.06) | 3.73 (1.07) | 3.86 (1.04) | -0.065, p=0.236 | -0.022, p=0.840 |
| Total score behavior *(max. 40 points)* | 31.23 (7.12) | 30.77 (7.26) | 31.54 (7.00) | 32.46 (5.83) | 31.89 (6.09) | 32.87 (5.61) | 1.235, p<0.001 | -0.216, p=0.722 |
| **Intentions** | | | | | | | | |
| How often do you intend to buy ready-to-eat chicken at an outlet that looks (visibly) clean? *(1=never, 5=always)* | 4.59 (0.75) | 4.57 (0.78) | 4.62 (0.73) | 4.52 (0.80) | 4.46 (0.85) | 4.56 (0.75) | -0.077, p=0.040 | -0.049, p=0.520 |
| How often do you intend to buy ready-to-eat chicken at an outlet where the vendor looks (visibly) clean? *(1=never, 5=always)* | 4.59 (0.76) | 4.53 (0.82) | 4.63 (0.72) | 4.54 (0.76) | 4.50 (0.80) | 4.56 (0.74) | -0.054, p=0.132 | -0.077, p=0.520 |
| How often do you intend to buy ready-to-eat chicken at an outlet where you can see that separate materials are used for preparing raw chicken and other prepared food? *(1=never, 5=always)* | 4.33 (1.03) | 4.30 (1.05) | 4.36 (1.01) | 4.40 (0.91) | 4.37 (0.94) | 4.42 (0.89) | 0.067, p=0.149 | 0.004, p=0.968 |
| How often do you intend to buy ready-to-eat chicken at an outlet where you can see that clean materials were used for preparing food? *(1=never, 5=always)* | 4.43 (0.92) | 4.40 (0.96) | 4.46 (0.90) | 4.47 (0.84) | 4.44 (0.90) | 4.49 (0.80) | 0.036, p=0.385 | 0.002, p=0.983 |
| How often do you intend to buy ready-to-eat chicken at an outlet that sells good quality chicken? *(1=never, 5=always)* | 4.73 (0.65) | 4.74 (0.63) | 4.72 (0.65) | 4.67 (0.67) | 4.60 (0.75) | 4.69 (0.62) | -0.070, p=0.028 | -0.110, p=0.091 |
| How often do you intend to wash your hands with clean water AND soap at the outlet? *(1=never, 5=always)* | 4.70 (0.74) | 4.69 (0.70) | 4.70 (0.77) | 4.62 (0.67) | 4.57 (0.71) | 4.66 (0.64) | -0.075, p=0.030 | -0.077, p=0.270 |

| | | | | | | | | |
|---|---|---|---|---|---|---|---|---|
| How often do you intend to check if your ready-to-eat chicken is served hot at the outlet? *(1=never, 5=always)* | 4.70 (0.66) | 4.67 (0.71) | 4.72 (0.62) | 4.61 (0.71) | 4.58 (0.69) | 4.63 (0.72) | -0.090, p=0.006 | -0.013, p=0.847 |
| How often do you intend to check that your ready-to-eat chicken is served on clean plates or containers that have been washed with clean water at the outlet? *(1=never, 5=always)* | 4.48 (0.88) | 4.40 (0.94) | 4.54 (0.83) | 4.59 (0.74) | 4.54 (0.76) | 4.63 (0.73) | 0.109, p=0.005 | 0.109, p=0.456 |
| **Mean score intentions (max. 5)** | 4.57 (0.61) | 4.54 (0.63) | 4.59 (0.60) | 4.55 (0.63) | 4.51 (0.66) | 4.58 (0.61) | -0.019 p=0.518 | -0.018, p=0.763 |
| **Knowledge** | | | | | | | | |
| What characterizes a visibly clean chicken outlet/koassa? *(max. 1)* | 0.29 (0.12) | 0.29 (0.12) | 0.30 (0.12) | 0.45 (0.17) | 0.46 (0.17) | 0.44 (0.16) | 0.156, p<0.001 | 0.024, p=0.099 |
| What characterizes a visibly clean vendor/koassa? *(max. 1)* | 0.33 (0.14) | 0.32 (0.13) | 0.56 (0.20) | 0.55 (0.20) | 0.54 (0.20) | 0.34 (0.14) | 0.218, p<0.001 | -0.012, p=0.480 |
| Please tell me if you think the following statement is true or false? "It is safe when a vendor uses the same materials such as cutting boards or knives for preparing raw chicken and then other food products" *(max 1)* | 0.64 (0.48) | 0.67 (0.47) | 0.63 (0.48) | 0.80 (0.40) | 0.78 (0.41) | 0.82 (0.39) | 0.158, p<0.001 | -0.078, p=0.062 |
| Please tell me if you think the following statement is true or false? "it is safe when a vendor uses the same unwashed knife he cut the raw chicken with for other food products" *(max. 1)* | 0.69 (0.46) | 0.71 (0.45) | 0.68 (0.47) | 0.88 (0.33) | 0.88 (0.33) | 0.88 (0.33) | 0.183, p<0.001 | -0.027, p=0.499 |
| How can you tell the ready-to-eat chicken you ordered at the outlet is of good quality? *(max. 1)* | 0.31 (0.23) | 0.29 (0.21) | 0.33 (0.23) | 0.29 (0.20) | 0.29 (0.21) | 0.29 (0.20) | -0.023, p=0.018 | 0.040, p=0.490 |
| When buying ready-to-eat chicken at the outlet, when should you wash your hands with water and soap? *(max. 1)* | 0.81 (0.39) | 0.86 (0.35) | 0.78 (0.41) | 0.71 (0.46) | 0.65 (0.48) | 0.74 (0.44) | -0.108, p<0.001 | -0.108, p<0.001 |
| When your ready-to-eat chicken is served at the outlet, how can you tell if the chicken meat has been cooked thoroughly? *(max. 1)* | 0.38 (0.20 | 0.36 (0.20) | 0.39 (0.20) | 0.50 (0.22) | 0.49 (0.22) | 0.51 (0.25) | 0.121, p<0.001 | 0.008, p=0.691 |
| **Total knowledge score *(max. 7)*** | 3.47 (0.94) | 3.51 (0.95) | 3.44 (0.94) | 4.17 (0.87) | 4.09 (0.85) | 4.23 (0.87) | 0.705, p<0.001 | -0.208, p=0.019 |
| **Risk perception** | | | | | | | | |
| To what extent do you think the ready-to-eat chicken you eat at the market is safe? *(1=very unsafe, 5=very safe)* | 2.54 (1.30) | 2.49 (1.30) | 2.58 (1.30) | 3.08 (1.29) | 3.04 (1.25) | 3.11 (1.31) | 0.535, p<0.001 | 0.016, p=0.875 |
| **Perceived health benefits** | | | | | | | | |
| Consistently paying attention to food safety behaviors when buying and consuming ready-to-eat chicken at the outlet is *(1=very detrimental, 5=very beneficial)* | 4.56 (0.65) | 4.62 (0.58) | 4.53 (0.69) | 4.44 (0.70) | 4.36 (0.73) | 4.49 (0.68) | -0.129, p<0.001 | -0.215, p<0.001 |
| **Self-efficacy** | | | | | | | | |

| | | | | | | | | |
|---|---|---|---|---|---|---|---|---|
| How confident are you that you can consistently pay attention to food safety behaviors when buying and eating ready-to-eat chicken at the outlet.: *(1=not at all, 5=very much)* | 3.87 (1 .07) | 3.81 (1.16) | 3.91 (1.01) | 4.09 (0.81) | 4.07 (0.84) | 4.11 (0. 79) | 0.217, p<0.001 | 0.062, p=0.496 |
| How difficult or easy do you think it is to consistently pay attention to food safety behaviours when buying ready-to-eat chicken at the market*: (1=very difficult, 5=very easy)* | 3.02 (1.34) | 2.98 (1.37) | 3.04 (1.32) | 3.36 (1.09) | 3.32 (1.09) | 3.39 (1.08) | 0.345, p<0.001 | -0.018, p=0.877 |
| **Perceived social (descriptive) norms** | | | | | | | | |
| People who are important to me consistently pay attention to food safety behaviors when buying ready-to-eat chicken at the outlet *(1=disagree, 5=agree)* | 3.28 (1.31) | 3.31 (1.29) | 3.25 (1.33) | 3.31 (1.07) | 3.27 (1.03) | 3.34 (1.10) | 0.034, p=0.528 | -0.135, p=0.219 |
| **Access to information** | | | | | | | | |
| To what extent can you access the information that you need for making informed decisions regarding the consumption of ready to eat chicken at the market? (none, some, most to all) | 2.36 (0.72) | 2.35 (0.72) | 2.37 (0.72) | 2.33 (0.66) | 2.28 (0.68) | 2.37 (0.64) | -0.026, p=0.316 | -0.071, p=175 |
| To what extent do you feel informed about food safety of ready-to-eat chicken meat in markets? *(1=not at all, 5=a lot)* | 2.94 (1.42) | 2.94 (1.45) | 2.96 (1.40) | 3.15 (1.20) | 2.99 (1.20) | 3.27 (1.20) | 0.205, p=0.001 | -0.259, p=0.044 |
